# Mitochondria: An Integrative Hub Coordinating Circadian Rhythms, Metabolism, the Microbiome, and Immunity

**DOI:** 10.3389/fcell.2020.00051

**Published:** 2020-02-07

**Authors:** Bruno A. Aguilar-López, María Maximina Bertha Moreno-Altamirano, Hazel M. Dockrell, Michael R. Duchen, Francisco Javier Sánchez-García

**Affiliations:** ^1^Laboratorio de Inmunorregulación, Departamento de Inmunología, Escuela Nacional de Ciencias Biológicas, Instituto Politécnico Nacional, Mexico City, Mexico; ^2^Department of Infection Biology, London School of Hygiene & Tropical Medicine, London, United Kingdom; ^3^Department of Cell and Developmental Biology, University College London, London, United Kingdom

**Keywords:** mitochondria, circadian rhythmicity, metabolism, intestinal microbiota, immune system

## Abstract

There is currently some understanding of the mechanisms that underpin the interactions between circadian rhythmicity and immunity, metabolism and immune response, and circadian rhythmicity and metabolism. In addition, a wealth of studies have led to the conclusion that the commensal microbiota (mainly bacteria) within the intestine contributes to host homeostasis by regulating circadian rhythmicity, metabolism, and the immune system. Experimental studies on how these four biological domains interact with each other have mainly focused on any two of those domains at a time and only occasionally on three. However, a systematic analysis of how these four domains concurrently interact with each other seems to be missing. We have analyzed current evidence that signposts a role for mitochondria as a key hub that supports and integrates activity across all four domains, circadian clocks, metabolic pathways, the intestinal microbiota, and the immune system, coordinating their integration and crosstalk. This work will hopefully provide a new perspective for both hypothesis-building and more systematic experimental approaches.

## Circadian Rhythmicity

Circadian rhythms were first observed in 1729 by Jean-Jacques d’Ortous de Mairan, who noticed that the leaves of the Mimosa plant moved with a periodicity of 24 h, even in the absence of light, thus suggesting the presence of an internal clock. It is now recognized that circadian rhythmicity integrates a mechanism for the timely coordination of cellular and broader physiological functions (Roenneberg and Merrow, 2005).

### The Circadian Clock

The term circadian is used to refer to biological cycles with a time length of about 24 h (Scheiermann et al., 2018), and the suprachiasmatic nucleus (SCN) is the “master clock” that coordinates and synchronizes those daily biological rhythms (Hastings et al., 2018).

Circadian rhythmicity is also regulated by a set of peripheral “clock proteins,” which form a hierarchy of oscillators that function at the cellular, tissue, and systems levels and are composed of at least three feedback loops (Mohawk et al., 2012; Curtis et al., 2014). One loop depends on the heterodimerization of the transcription factors brain and muscle aryl hydrocarbon receptor nuclear translocator-like 1 (BMAL1) and circadian locomotor output cycles kaput (CLOCK) that, upon binding to E-box elements, induce the expression of their own repressors, named Period (PER) and Cryptochrome (CRY) proteins. Since these proteins (PER-1, -2, and -3, and CRY-1 and -2) are gradually degraded, the expression on BMAL1 and CLOCK ceases, starting a new circadian cycle.

A second loop is formed by the nuclear retinoic acid receptor-related orphan receptor (ROR) (α, β, γ) and REV-ERB (α, β), which, upon activation by the BMAL1/CLOCK heterodimer and translocation into the nucleus, bind to receptor-related orphan receptor response elements (ROREs) in the promoter of BMAL1, regulating the expression of BMAL1 (Mohawk et al., 2012; Curtis et al., 2014).

**FIGURE 1:**
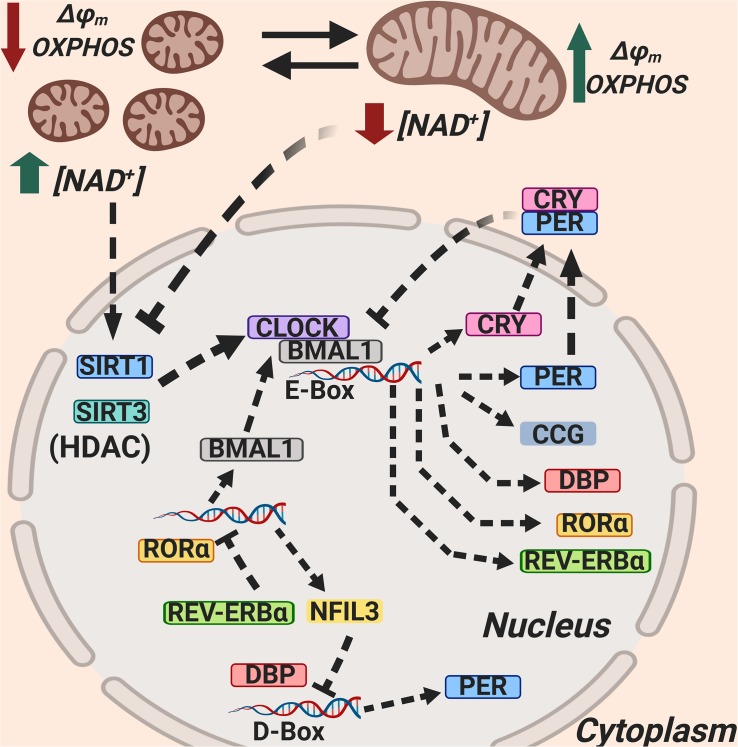
Mitochondria regulate circadian rhythmicity through NAD^+^ production, SIRT1 and SIRT3 activation, and mitochondrial dynamics. The Sirtuin 1 (SIRT1) and Sirtuin 3 (SIRT3) activity as HDACs is dependent on NAD^+^, and SIRT1 and SIRT3 counteract CLOCK; NAD^+^ synthesis is dependent on circadian rhythmicity, and this is related to mitochondrial dynamics. NAD^+^, Nicotinamide adenin dinucleotide (oxidized); SIRT1, Sirtuin 1; SIRT3, Sirtuin 3; BMAL1, Brain and muscle aryl hydrocarbon receptor nuclear translocator-like 1; CLOCK, circadian locomotor output cycles kaput; RORα, Retinoic acid receptor-related orphan receptor α; REV-ERBα, Reverse strand of ERBA (REV-ERBα is encoded by the opposite DNA strand of the ERBA oncogene, hence its name); NFIL3, nuclear factor interleukin 3; DBP, D-box binding protein; CRY, Cryptochrome; PER, Period; CCG, Clock-Controlled Genes.

A third loop is formed by the transcriptional activator albumin D-box binding protein (DBP) and the repressor nuclear factor interleukin 3 (NFIL3), which synergistically regulate the expression of D-box genes, including the Per genes. The interplay between these three regulatory loops is at the core of circadian rhythmicity and clock-related gene expression (Mohawk et al., 2012; Curtis et al., 2014; Figure 1).

### Circadian Rhythmicity and Its Physiological Role

Circadian rhythms modulate nearly every mammalian physiological process, including sleep, feeding times, energy metabolism, endocrine, and immune functions. Genome-wide transcriptional profiling analyses have shown that up to 35–50% of the genome displays rhythmic expression in eukaryotes (Shi and Zheng, 2013). Light acts as a circadian cycle activator that helps to regulate the peaks of physiological functions such as rest-activity, respiration, blood pressure, and body temperature (Zhang et al., 2014).

Circadian rhythms also regulate metabolism, and so there is a differential circadian-based expression of glucose transporters, glucagon receptors, and glycolysis-related enzymes and levels of insulin, glucagon, leptine, and cortisone (Panda, 2016), as well as circadian-based differences in the absorption, transport, and serum levels of cholesterol, triglycerides, and apolipoproteins (Poggiogalle et al., 2018).

Several immune functions are under direct control of the circadian clock, such as leukocyte traffic (Gibbs et al., 2014; Druzd et al., 2017), the production of cytokines, granzymes, and perforins (Logan and Sarkar, 2012), phagocytosis and bactericidal activities (Casanova-Acebes et al., 2013; Oliva-Ramírez et al., 2014), expression of diverse pattern recognition receptors (PRRs) (Silver et al., 2018), response to pathogens (Kiessling et al., 2017; Zhuang et al., 2017), anti-inflammatory responses (Gibbs et al., 2014), T lymphocyte responses (Fortier et al., 2011), and allergic responses (Paganelli et al., 2018).

In addition, intestinal bacteria display endogenous circadian rhythmicity and influence the function of the intestinal circadian clock, and the host circadian rhythmicity influences the composition of bacterial communities in the intestine (Asher and Sassone-Corsi, 2015; Paulose et al., 2016; Huang et al., 2018).

### Pathological Consequences of Disrupting the Circadian Rhythmicity

The incidence of pathologies such as depression, obesity, diabetes, infarction, cancer, and others is higher in time-shift workers, and a common risk factor is inflammation (Castanon-Cervantes et al., 2010; Schwartsburd, 2017). Shift work, leading to a disruption in circadian rhythmicity, has been classified within the group 2A of probable human carcinogens (Straif et al., 2007). Long flights that result in jet lag, night work, and exposure to artificial light during the night can also disrupt circadian rhythmicity, leading to irritability, anxiety, and depressive behavior (Salgado-Delgado et al., 2011). In this regard, animal models of light-dark cycle-controlled changes have contributed to our understanding of how circadian rhythmicity correlates with physiological functions such as metabolism and cognitive processes (Fujioka et al., 2011).

In many immunological and allergic diseases, including rheumatoid arthritis, bronchial asthma, atopic eczema, and chronic urticarial irritation, the intensity of symptoms and disease severity show a circadian pattern (Nakao et al., 2015; Stull et al., 2017).

### Mitochondrial Regulation of Circadian Rhythmicity

Mitochondria constantly undergo changes in both morphology and distribution within the cytoplasm, as fused (network forming) or fissioned (punctate) mitochondria, a process collectively referred to as mitochondrial dynamics; fused mitochondria are regarded as metabolically more active than fragmented mitochondria, as cells with a fused mitochondrial network seem to have a higher respiratory rate than cells with fragmented mitochondria (Westermann, 2012; Mishra and Chan, 2016). Likewise, it has been suggested that there is a correlation between circadian rhythmicity and mitochondrial function (Kohsaka et al., 2014; Oliva-Ramírez et al., 2014; Scrima et al., 2016; de Goede et al., 2018; Ezagouri and Asher, 2018).

Moreover, cells with disrupted mitochondria, such as Rho 0 cells, lack a well-defined circadian rhythmicity, in part due to the lack of the characteristic robust oscillatory respiratory activity observed in cells with healthy mitochondria (Scrima et al., 2016). Mechanistically, the mitochondrial fusion-fission process is dependent, amongst other proteins, on dynamin-related protein-1 (Drp1) and its phosphorylation state; phosphorylated Drp1 promotes mitochondrial fusion, and Drp1 is phosphorylated in a circadian-dependent manner, thus varying its activity according to light/dark cycles (Schmitt et al., 2018).

The circadian clock also rhythmically regulates the biosynthesis of NAD^+^ and, in this way, the mitochondrial capacity for energy production. Mitochondrial NAD^+^ also determines the activity of the deacetylases SIRT1 and SIRT3, which in turn control the acetylation and activity of other key metabolic enzymes. Interestingly, as an example of bi-directional communication, NAD^+^ also influences clock function (Belden and Dunlap, 2008; Peek et al., 2013; Figure 1).

## Metabolism

Metabolism, broadly defined as the sum of biochemical processes in living organisms that produce or consume energy, is at the core of many human diseases, and the current view is that metabolism is not just a self-regulating network but one that impacts, or is impacted by, many other cellular processes (DeBerardinis and Thompson, 2012).

### Metabolic Pathways

Recent comprehensive reviews have described glycolysis, Krebs cycle, pentose phosphate pathway, fatty acid oxidation, fatty acid synthesis, and amino acid metabolic pathways and how these play key roles in immune cell effector functions (O’Neill et al., 2016; Geltink et al., 2018; Russell et al., 2019), highlighting the close connection between metabolism and the immune system (immunometabolism). A detailed description of these metabolic pathways is out of the scope of this review. Rather, in order to get some insight into the relationship between metabolism and the other three biological domains here referred to, we discuss, in the next two sections, the role, beyond metabolism, of specific eukaryotic metabolites, and briefly consider the concept of the metabolome.

### Beyond Metabolism: Non-canonical Biological Roles of Some Eukaryotic Metabolites

#### Lactate

Lactate is a natural ligand for the GPR81 cell membrane receptor that also recognizes other monocarboxylates; lactate enhances cell differentiation, suppresses T-cell proliferation, reduces the cytotoxic capacity of cytotoxic T lymphocytes, stimulates gene expression, and plays an important role in the tumor microenvironment (Mosienko et al., 2015; Romero-García et al., 2016; Luo et al., 2017).

**FIGURE 2:**
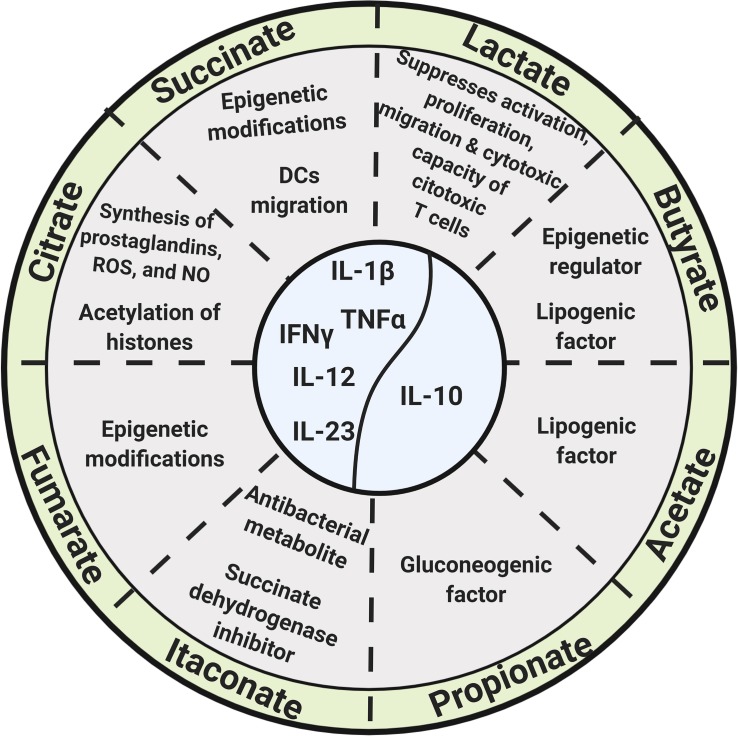
Glycolysis and Krebs cycle-derived metabolites, as well as microbiota-derived metabolites, exert biological functions beyond energetic and biosynthetic metabolism.

Extracellular lactate concentrations in the cerebral cortex vary during the day (Naylor et al., 2012), and Bmal1, a clock gene, regulates the expression of pyruvate kinase M2 (PKM2) and hence lactate production, which is required for the expression of the immune checkpoint protein PD-L1 (programmed cell death ligand 1) in tumor cells and immune cells (Palsson-McDermott et al., 2015; Deng et al., 2018; Figure 2).

#### Citrate

Citrate is exported from mitochondria to the cytosol by means of the mitochondrial citrate transporter; once in the cytosol, citrate is converted to acetyl-CoA and oxalacetate by the ATP-citrate lyase, and acetyl-CoA promotes the acetylation of histones and the promoter regions of TNF-α and IL-8 genes, increasing the production of IL-1β, TNF-α, and IFNγ (Ashbrook et al., 2015; Mills et al., 2017).

#### Itaconate

Itaconate results from the conversion of citrate to cis-aconitate and then to itaconate by the extramitochondrial enzyme cis-aconitate decarboxylase in a “break point” of the Krebs cycle during macrophage transition to a proinflammatory state. It is one of the most highly induced metabolites in activated macrophages and acts as an antibacterial metabolite by inhibiting the bacterial enzyme isocitrate lyase (Rittenhouse and McFadden, 1974; Garaude et al., 2016; Lampropoulou et al., 2016; Belizário et al., 2018; Figure 2). The enzyme cis-aconitate decarboxylase is encoded by the immune-responsive gene 1 (Irg1) (Michelucci et al., 2013); expression of Irg1 in macrophages is required for ATP and GTP synthesis (Németh et al., 2016; Belizário et al., 2018).

Itaconate functions as an endogenous succinate dehydrogenase (SDH) inhibitor, and, therefore, its production or its exogenous addition (or its derivatives) regulates succinate levels, as well as mitochondrial respiration and inflammatory cytokine production (Cordes et al., 2016; Lampropoulou et al., 2016; Mills et al., 2017; Belizário et al., 2018). The addition of itaconate or dimethyl itaconate to macrophages reduces the expression of pro-IL-1β, IL-6, IL-12, and iNOS (Lampropoulou et al., 2016; Belizário et al., 2018), and it is also possible that itaconate modifies cysteine residues of multiple target proteins, contributing to its role as an immunomodulator, as pointed out by Hooftman and O’Neil in a recent review (Hooftman and O’Neill, 2019; Figure 2).

#### Succinate

Succinate is produced from succinyl CoA by succinyl-CoA synthetase and accumulates in the cytoplasm of monocyte/macrophages and dendritic cells (DCs) upon LPS stimulation (Pistollato et al., 2010; Williams and O’Neill, 2018). Succinate accumulation, along with the induction of the glycolytic enzyme hexokinase-1, increases the activity of the respiratory chain complex II, promotes the production of mROS, stabilizes HIF-1α, regulates the transcription of pro-IL-1β, and activates the NOD-like receptor protein 3 (NLRP3) inflammasome, increasing the production of IL-1β (Chandel et al., 2000; Moon et al., 2015; Garaude et al., 2016; Mills et al., 2016, 2017; Figure 2).

LPS stimulation leads to the succinylation of several enzymes such as glyceraldehyde 3-phosphate dehydrogenase (GAPDH), malate dehydrogenase (MDH), lactate dehydrogenase (LDH), and the glutamate carrier-1 (Tannahill et al., 2013).

A specific receptor for extracellular succinate (SUCNR1/GPR91) is present in hepatic, renal, retinal, and immune cells, and its ligation leads to the secretion of various hormones, growth factors, and cytokines (Ariza et al., 2012) and regulates DCs migration into lymph nodes, as well as DCs antigen presentation (Figure 2). The SUCNR1/GPR91 receptor can synergize with TLR3 and TLR7, increasing the production of pro-inflammatory cytokines (Rubic et al., 2008).

Succinate is also a competitive inhibitor of multiple α-ketoglutarate (α-KG)-dependent dioxygenases, including histone demethylases and prolyl hydroxylases, thus contributing to epigenetic regulation (Xiao et al., 2012; Figure 2).

#### Fumarate

Fumarate is produced by the oxidation of succinate by the SDH enzyme (respiratory complex II) (Cecchini, 2003). Fumarate and its derivatives have strong bacteriostatic and bactericidal activity (Garaude et al., 2016); dimethyl fumarate (DMF), through its metabolite monomethylfumarate (MMF), is also a potent immunomodulator and antioxidant (Wilms et al., 2010; Cross et al., 2011). DMF induces DCs to produce IL-10, IL-12, and IL-23, tuning-down pathogenic T lymphocytes (Schlöder et al., 2017).

Dimethyl fumarate inhibits maturation of DCs (Peng et al., 2012), Th1 to Th2 lymphocyte shift (Wu et al., 2017), pro-inflammatory cytokine signaling (McGuire et al., 2016), nuclear translocation of NF-κB (Gillard et al., 2015), and the expression of cell adhesion molecules in lymphocytes and endothelial cells (Rubant et al., 2008). DMF has anti-inflammatory activity on murine astrocytes by activating the Nrf2 transcription factor, reducing oxidative stress and increasing *in vivo* neuroprotection (Linker et al., 2011); hence, its therapeutic use in patients with neurological diseases such as multiple sclerosis (Wingerchuk and Carter, 2014).

Fumarate accumulates in macrophages in the course of β-glucan-induced innate immune training, and, strikingly, the addition of exogenous fumarate to macrophages *in vitro* induces innate immune training concomitant to the induction of an epigenetic landscape similar to that of β-glucan-induced training (Arts et al., 2016; Figure 2).

### The Metabolome

The metabolome is the repertoire of small biomolecules present in cells, tissues, and body fluids, and its composition is at the core of the health status of individuals. The development of new “metabolomic platforms” has revealed that a number of metabolites present in several biological samples, such as serum and urine, vary in concentration following a circadian rhythmicity (Martínez-Lozano et al., 2014; de Raad et al., 2016). Among them are glycolysis-related metabolites, such as glucose, glucose-6-phosphate, bisphosphoglycerate, and lactate; tricarboxylic acid (TCA) cycle-related molecules, such as acetate, acetyl CoA, citrate, isocitrate, and malonate; amino acids and their derivatives; lipid metabolites; nucleotides; antioxidants; and coenzymes such as NAD, FAD, and coenzyme A (Krishnaiah et al., 2017).

Interestingly, the daily variation in the bacterial composition within the intestine implies a daily variation in the concentration of some bacteria-derived metabolites, and the hundreds of microbiota-derived metabolites that have been identified are regarded as components of the human metabolome (Belizário et al., 2018). Thus, linking eukaryotic- and bacterial-derived metabolites with the other three biological domains is discussed here.

In attempting to convey the view that mitochondria support and integrate the communication between the four mentioned biological domains, the specific roles of mitochondria are discussed in the next sections.

### Mitochondria as a Metabolic Hub

Mitochondria are at the core of metabolic pathways. They produce most of the energy supply for cells by means of oxidative phosphorylation coupled to the electron transport chain (ETC); complete oxidation of glucose by cells yields up to 33.45 ATP molecules from each molecule of glucose (Mookerjee et al., 2017).

Mitochondria also participate in the synthesis of fatty acids, metabolic intermediates, amino acids, and reactive oxygen species (ROS) (Spinelli and Haigis, 2018) and the maintenance of the cellular redox state and function as a signaling platform in innate immunity (Weinberg et al., 2015).

The bioenergetics status of mitochondria also appears to be regulated by a fission-fusion process. Mitochondrial fission is regulated by the action of Drp1, mitochondrial fission factor (Mff), mitochondrial fission protein 1 (Fis1), MiD49, and MiD50; the assembly of Drp1 proteins constricts the mitochondria, breaking apart sections of them, downregulating OXPHOS constituents (Chan, 2012; Labbé et al., 2014).

Mitochondrial fusion is controlled by GTPases of the dynamin superfamily, such as mitofusin 1 and mitofusin 2 (Mfn1 and Mfn2) and optic atrophy 1 (Opa1), and this process increases OXPHPOS (Chen et al., 2010; Cogliati et al., 2013).

Mitochondria take up calcium, which enables the modulation of Ca^2+^ levels and Ca^2+^ signaling in their immediate proximity. In addition, Ca^2+^ uptake by mitochondria stimulates the TCA cycle and oxidative phosphorylation (Duchen, 1992; Wang et al., 2019). The activity of several bioenergetics-related enzymes, such as glycerol phosphate dehydrogenase, pyruvate phosphate dehydrogenase, isocitrate dehydrogenase, oxoglutarate dehydrogenase, SDH, and NADH dehydrogenase, are regulated by calcium (Panov and Scaduto, 1995; Huang et al., 1998).

Moreover, mitochondria can be transferred from one cell to another, and, thus, injured cells can receive mitochondria from healthy cells, enhancing their cellular bioenergetics, and can even improve organ function, such as in acute lung injury and other inflammatory diseases (Islam and Luster, 2012). Several mechanisms may account for the intercellular transfer of mitochondria (Torralba et al., 2016), including tunneling nanotubes (Jackson et al., 2016), direct cytoplasmic transfer (Spees et al., 2006), extracellular vesicles (Spees et al., 2006), and micropinocytosis (Kitani et al., 2014).

## Intestinal Microbiota

Humans are colonized in diverse anatomical sites by a myriad of commensal microorganisms, collectively referred to as the microbiota (the microbial taxa associated with humans) or as the microbiome (the catalog of these microbes and their genes), an important component of which is the intestinal microbiota, which has profound effects on the host physiology (Ursell et al., 2012; Butler et al., 2019).

### Bacterial Colonization of the Intestinal Tract

Around 10^13^–10^14^ bacteria, from more than one thousand different species, colonize the human intestinal tract, and different anatomical regions within the intestine harbor distinctive microbial consortia or “microbiota” (Spor et al., 2011; Lozupone et al., 2012).

Fetuses are in contact with microorganisms within the uterus, and distinctive microbiomes have been found in the amniotic cavity, umbilical cord, and placenta; these microorganisms are not pathogenic, belong to the Firmicutes, Tenericutes, Proteobacteria, Bacteroidetes, and Fusobacteria phyla, and are perhaps the first bacteria to colonize the fetal gastrointestinal tract (Satokari et al., 2009; Aagard et al., 2014).

Within the first days of life, the intestine is colonized, mainly by aerobic microorganisms, and colonization reaches its maximal density within 72 h after birth; the phylogenetic diversity of microbiota gradually increases and allows colonization by anaerobic bacteria (Spor et al., 2011; Lozupone et al., 2012; Matamoros et al., 2013; Fulde and Hornef, 2014). Three years after birth, the intestinal microbiota has developed into a “mature” stage, characterized by its stability (Lozupone et al., 2012; Matamoros et al., 2013).

Newborns are exposed to another variety of microorganisms at the time of delivery; the vaginal route of delivery is associated with bacteria from Actinobacteria, Bacteroidetes, and Firmicutes phyla colonizing the newborn intestine (Koleva et al., 2015), whereas, in infants born by the cesarean procedure, the intestine is colonized by *Staphylococcus* spp. and *Streptococcus* from the mother’s skin (Dominguez-Bello et al., 2010).

Infants fed exclusively on maternal milk exhibit higher amounts of aerobic bacteria from the Bifidobacterium and Lactobacillus genera and a lower amount of strict anaerobic bacteria, such as *Clostridium difficile*, or facultative anaerobic bacteria, such as *Escherichia coli* (Koleva et al., 2015), whereas infants fed exclusively on formula are colonized by *C. difficile*, *Bacteroides*, and *Veillonella* species (Wang et al., 2015).

### Biological Role of Intestinal Microbiota

**FIGURE 3:**
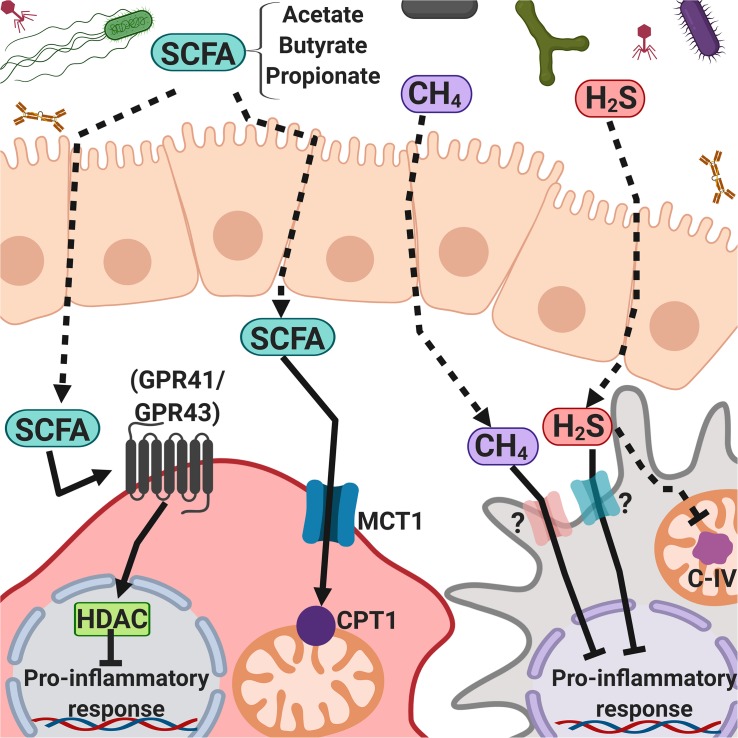
Intestinal microbiota-derived short-chain fatty acids, methane, and hydrogen sulfide regulate inflammatory responses. Microbiota-derived products such as SFCAs (mainly acetate, butyrate, and propionate), CH_4_, and H_2_S can cross the intestinal epithelial cell barrier and interact with the immune system cells, activating G protein-coupled receptors or passing through cell membranes, promoting cell signaling and modulating the immune response.

The commensal intestinal microbiota is a key determinant for human health; it contributes to host digestive processes (Lozupone et al., 2012) and is a source of folate and vitamins A and B, which regulate host chromatin-modulating enzymes (Qin and Wade, 2018), and several amino acids, including glycine, which is required for the synthesis of glutathione, the main intracellular antioxidant and detoxifying molecule (Belizário et al., 2018). It also stimulates intestinal immune responses by contributing to the development of gut-associated lymphoid tissues (GALT), especially at early stages of life, produces short-chain fatty acids (SCFAs), such as butyrate, acetate, and propionate, that have immunomodulatory properties, and regulates local immune responses (Figure 3). The intestinal microbiota also suppresses pathogens through the production of bactericidal proteins and prevents the intestine from colonization by pathogenic bacteria (Ashida et al., 2011; Lozupone et al., 2012; Kamada et al., 2013; Kabat et al., 2014; Rescigno, 2014).

Intestinal bacteria produce large amounts of methane, hydrogen sulfide, and non-gaseous metabolites, some of which have signaling properties that turn on and off an array of host genes, as well as virulence and metabolism-related microbial genes (Tremaroli and Bäckhed, 2012; Belizário et al., 2018; Rowland et al., 2018; Figure 3).

### Dysbiosis

Change in the composition of intestinal microbiota, both commensal and pathogenic, is known as dysbiosis; this condition may affect homeostasis, leading to non-specific inflammation and disease. Dysbiosis implies an imbalance in microbial metabolite composition (Belizário et al., 2018) and is mainly the result of an “unhealthy” diet, the use of antibiotics, and lifestyle factors (Dudek-Wicher et al., 2018), it can also be caused by emotional and physiological stress (Li et al., 2018). Dysbiosis may result in epigenetic changes in adjacent intestinal cells, as well as in hepatocytes and adipocytes (Belizário et al., 2018; Qin and Wade, 2018).

Rapid changes in feeding habits over the last century may have contributed to our current enterotypes and general health (Moeller et al., 2014). Bowel disease, irritable bowel syndrome, obesity, diabetes, and cancer have been associated with specific bacterial dysbiosis (Clemente et al., 2012; Belizário et al., 2018). Infants born by Cesarean delivery or from mothers that have used antibiotics and thus harbor a particular enterotype, have a higher risk of developing asthma, type I diabetes, and celiac disease (Mueller et al., 2015).

There is evidence of a time-of-day-specific intestinal microbiota taxonomic composition associated with rhythmic food intake, dietary structure, gender, and the host biological clock (Liang et al., 2015; Thaiss et al., 2016; Li et al., 2018), and there is a clear role for the intestinal microbiota in the regulation of metabolism, the immune system, and circadian rhythmicity. One of the mechanisms of communication between the intestinal microbiota and the other three biological domains involves microbial metabolites.

### Microbial Metabolites

Intestinal microbiota-derived metabolites have a major influence on host physiology, particularly SCFAs, as well as hydrogen sulfide (H_2_S) and methane (CH_4_). SCFAs are produced from larger fatty acids and from the microbial catabolism of carbohydrates; and H_2_S and CH_4_, act as messengers to colonic epithelial and immune cells, impacting their metabolism, epigenetics, and gene expression.

A higher proportion of SCFA-producing bacteria within the intestinal microbiota is associated with a reduction in the risk of developing obesity, insulin resistance, and type 2 diabetes, since these compounds, particularly butyrate, increase cellular respiration and fatty acid oxidation (Belizário et al., 2018). Acetate, butyrate, and propionate are the most abundant SCFAs and represent 90–95% of the total SCFAs present in the colon, at physiological concentrations in the order of 50–100 nM (Ríos-Covián et al., 2016).

Short-chain fatty acids are ligands of the free fatty acid receptor 2 (also called GPR41) and the free fatty acid receptor 3 (also called GPR43) that, upon activation in intestinal L cells, induce the release of glucagon-like peptide-1 (GLP-1), which contributes to insulin signaling in white adipocytes, reducing adiposity (Greiner and Bäckhed, 2016; Ang et al., 2018). Propionate is a gluconeogenic factor, whereas acetate and butyrate are lipogenic factors (Belizário et al., 2018; Rowland et al., 2018).

Butyrate and its structural analog, β-hydroxy-butyrate, act as histone deacetylases (HDACs) and therefore play a role in epigenetic regulation (Shimazu et al., 2013) and exert a protective effect on the fatty liver, oxidative stress, and mitochondrial dysfunction associated with obesity and insulin resistance (Mollica et al., 2017).

Butyrate downregulates the expression of co-stimulatory molecules, the maturation, metabolic reprograming, and secretion of IL-12, IL-6, and NO in monocytes/macrophages and DCs, inhibits the nuclear translocation of NF-κB, and drives T cells to produce IL-10 and IL-17; propionate inhibits the secretion of IL-12 and IL-33 by LPS-activated DCs, reduces the expression of co-stimulatory molecules on DCs, and inhibits cytotoxic T lymphocytes (Berndt et al., 2012; Chang et al., 2014; Kaisar et al., 2017; Nastasi et al., 2017). Oral administration of sodium butyrate increases the percentage of CD4^+^ Foxp3^+^ Tregs in the colon but not in lymph nodes and spleen, and this correlates with the increase in IL-10 in the intestine (Kespohl et al., 2017; Figure 3).

Hydrogen sulfide and methane help to modulate the circulatory, nervous, and immune systems, as well as endothelial cells (Boros et al., 2015). The plasma concentration of H_2_S is lower than 1 μM (Shen et al., 2012), and the mechanism proposed for its biological activity is protein modification by S-sulfhydration of cysteine residues. H_2_S has a dual activity, acting both as an anti-inflammatory and as a pro-inflammatory molecule that regulates T and B lymphocytes, NK cells, basophils, and monocytes (Sulen et al., 2016; Bourque et al., 2018). H_2_S reacts with heme protein groups, acting as a competitive inhibitor of mitochondrial complex IV (Figure 3). However, at low concentrations, this gas increases cellular respiration (Ming et al., 2012; Nicholls et al., 2013).

In macrophages, the exposure to CH_4_ (as CH_4_-rich saline, 0.99 mmol/L) leads to the activation of GSK-3β, which attenuates the phosphorylation of NF-κB and MAPK, negatively regulating the production of pro-inflammatory cytokines both *in vitro* and *in vivo* (Zhang et al., 2016; Figure 3). As mentioned in section “Metabolome,” these microbial metabolites (acetate, butyrate, propionate, hydrogen sulfide, and methane), among many others, are components of the human metabolome.

### Mitochondria and Bacteria

Mitochondria have a bacterial origin, and thus it is likely that bacterial products may directly interact with mitochondria, modifying their function.

Mitochondrial metabolic stress induces mitochondrial dysfunction, which may lead to the disruption of the intestinal epithelial barrier, allowing *E. coli*, and perhaps other bacteria, to cross the epithelium (Nazli et al., 2004; Wang et al., 2015). Microbial products, such as butyrate and urolithin A, enhance mitochondrial functions (Ryu et al., 2016), and others, such as betaine, methionine, and homocysteine, activate signaling pathways that regulate mitochondrial dynamics in the intestinal epithelium (Lin and Wang, 2017). *E. coli*-secreted colanic acid is endocytosed and is capable of inducing Drp1-dependent mitochondrial fission (Han et al., 2017), and *Pseudomonas aeruginosa* secretes N-(3-oxo-dodecanoyl)-L-homoserine lactone (3OC12), a molecule thought to subvert immune defenses. In several cell types, such as in bronchial epithelial cells, 3OC12 is hydrolyzed by the enzyme lactonase paraoxonase 2 (PON2) present in mitochondria, yielding 3OC12 acid, which accumulates within mitochondria, causing mitochondrial and cytosolic acidification, increase in intracellular Ca^2+^ concentration and activation of stress signaling kinases (Horke et al., 2015).

Polymorphisms in the mitochondrial genes of the ND5, CYTB, and D-loop regions have been associated with variations in the composition of the intestinal microbiota (Ma et al., 2014); mutations in the ATP8 gene increase the relative abundance of Bacteroidales, Deferribacteraceae, Desulfovibrionaceae, and Helicobacteraceae, suggesting that mitochondria play a role in defining the microbiome (Hirose et al., 2017).

It has also been suggested that mitochondria from intestinal cells are highly responsive to microbiotic signaling, with implications in inflammatory processes and colorectal cancer (Andersson et al., 1998; Jackson and Theiss, 2019). In addition, itaconate, which is synthetized from the Krebs cycle aconitate, limits bacterial growth by inhibiting bacterial isocitrate lyase (Rittenhouse and McFadden, 1974). Whether itaconate has a role in shaping intestinal microbiota remains to be investigated.

## The Immune System

The immune system has long been regarded as a mechanism for self and non-self discrimination and, according to Janeway (2001), this distinction is virtually always made by the innate immune system, which primes the adaptive immune system. New evidence indicates that in addition to the crosstalk between the innate and adaptive immune systems, the immune system, as a whole, communicates with other biological domains, as outlined in the next sections.

### Circadian Rhythmicity and the Immune Response

Circadian rhythms regulate innate and adaptive immunity, influencing the outcome of infectious and immune system-related diseases (Scheiermann et al., 2013, 2018; Li et al., 2017). High-throughput analysis has revealed rhythmicity in more than 8% of the macrophage transcriptome, including many important regulators for pathogen recognition and cytokine secretion (Keller et al., 2009), and migration to inflamed or infected tissues, cytolytic activity, and proliferative response to antigens seem to be circadian clock-dependent (Labrecque and Cermakian, 2015).

The total numbers of hematopoietic stem cells and mature leukocytes reach their peak in circulation during the night and decrease during the day (Scheiermann et al., 2013), and the concentrations of cytokines and chemokines, such IL-6, TNF-α, and CXCL 12, also undergo daily around-the-clock fluctuations (Cermakian et al., 2013; Li et al., 2017). Clock genes and micro RNAs (miRs) participate in the circadian control of immune responses (Curtis et al., 2014, 2015).

The magnitude of the immune response to infectious agents and their products, such as gram-negative bacteria-derived lipopolysaccharides, is dependent on the time of the day of exposure (Bellet et al., 2013); it has been proposed that circadian rhythmicity allows anticipation of possible exposure to pathogenic agents, as well as more efficient use of the energy required to maintain the immune system (Scheiermann et al., 2013, 2018).

In a mouse model of multiple sclerosis (experimental autoimmune encephalomyelitis), the loss of the transcription factor BMAL1, a core component of the molecular clock, or the administration of the autoimmunity-eliciting antigen (MOG35–55 peptide) at midday rather than at midnight causes more severe immunopathology (Sutton et al., 2017), all of which highlights the importance of the circadian cycle to the immune system.

### Metabolic Regulation of the Immune Response

In 2002, a pioneering study demonstrated that, upon activation, T lymphocytes rely on a “Warburg-type” metabolism (aerobic glycolysis) and, remarkably, that immune co-stimulation is, in fact, a metabolic rewiring of the cell (Frauwirth et al., 2002). Since then, knowledge on the metabolic requirements for the immune cell effector functions to take place, as well as the immunomodulatory roles of metabolic intermediates, has grown steadily. It is currently known that, for instance, “classically activated” macrophages (LPS plus IFN-γ), also referred to as M1 macrophages, use the Warburg-type metabolic pathway, whereas alternatively activated macrophages (IL-4), also referred to as M2 macrophages, use OXPHOS and β-oxidation to generate energy (Rodríguez-Prados et al., 2010; O’Neill and Pearce, 2016; Mills et al., 2017). The antimicrobial function of macrophages is regulated by metabolic reprogramming that includes succinate accumulation (Tannahill et al., 2013), mitochondrial ROS production (West et al., 2011), and mitochondrial respiratory-chain adaptation (Garaude et al., 2016). Stimulation of B lymphocytes induces glycolysis and oxidative phosphorylation, which facilitates the production of IgG or IgA antibodies (Pearce and Pearce, 2018), and shifts in cell metabolism play a central role in T-cell quiescence, memory, and activation responses (Pearce and Pearce, 2018).

In addition, some metabolic intermediates contribute to delineating the immune response (Rodríguez-Prados et al., 2010; Arts et al., 2016). Collectively, this field of research is now referred to as “immunometabolism” (O’Neill et al., 2016).

### Intestinal Microbiota and the Immune Response

A driving force for the evolution of the immune system is perhaps the need to maintain a homeostatic host/microbiota interaction, shaping both host immunity and microbial ecology. The intestinal microbiota helps to shape the immune system, especially in the early stages of life, whereas microbiota composition may be related to susceptibility to immune system-related diseases in adulthood (Lozupone et al., 2012; Kabat et al., 2014).

The intestinal microbiota contributes to the intestinal immune responses, partly by their metabolites, which may activate regulatory T cells or support systemic anti-viral immunity, as in the case of the microbial metabolite desaminotyrosine, which promotes the expression of interferon-stimulated genes (Steed et al., 2017: Pearce and Pearce, 2018).

The immune system may shape the intestinal microbial composition by means of sensors of the innate immune system, such as Toll-like receptors (TLRs), Nod-like receptors (NLRs), peroxisome proliferator-activated receptors (PPARs), and aryl hydrocarbon receptors (AhR), and, perhaps, by humoral immune responses (Zelante et al., 2013; Kato et al., 2014).

### Mitochondria and the Immune Response

In addition to their role as the “power-house” of the cell, mitochondria have an active role in the immune response. Activation of the innate immune response through the engagement of PRRs by pathogen-associated molecular patterns (PAMPs) or damage-associated molecular patterns (DAMPs) turns on signaling pathways that lead to the synthesis of pro-inflammatory cytokines, and this process involves mitochondria for inflammasome activation; the mitochondrial components mtDNA and mROS activate the NLRP3 inflammasome, promoting the maturation of IL-1β and IL-18 from their pro-IL-1β and pro-IL-18 precursors (Zhou et al., 2011).

Mitofusin 2, one of the proteins that promote mitochondrial fusion, participates in the activation of the inflammasome, and elimination of Drp1, a protein that promotes mitochondrial fission, results in abnormal mitochondrial fusion, leading to increased activation of NLRP3-dependent activation of caspase 1 and IL-1β and IL-18 synthesis (Park et al., 2015).

Upon microbe phagocytosis by macrophages, phagosomes bind to lysosomes and to mitochondria, allowing the interchange of ions, amino acids, and lipids, as well as the acidification of the phago-lysosome; mutation of cardiolipin synthase 1 (Cdr1) decreases the levels of mitochondrial cardiolipin, lysosome acidification, and mitophagy; inhibition of OXPHOS or deletion of mitochondrial function-related proteins, such as Aif, Opa1, and PINK1, impairs lysosomal activity (Demers-Lamarche et al., 2016).

In the course of immune responses against RNA viruses, infected cells sense virus-related genetic material in their cytoplasm by means of the RIG-I-MDA5-mitochondrial antiviral-signaling protein (MAVS), which is the major sensing pathway for RNA viruses (Wu and Chen, 2014; Moreno-Altamirano et al., 2019); activation of MAVS is dependent on mitochondrial membrane potential (Δψm), and the coordination between MAVS signaling and mitochondrial fission increases the production of IFN-β (Koshiba, 2013).

Mitochondrial function is required for ROS production, autophagy, and antigen processing and presentation (Bonifaz et al., 2015; Oberkampf et al., 2018; Gómez-Cabañas et al., 2019). Mitochondria accumulate at the immune synapse in T lymphocytes (Quintana et al., 2007), and effector T lymphocytes are characterized by having fused mitochondria (Buck et al., 2016).

All this highlights the key role played by mitochondria in the immune response, and consequently, that alterations in mitochondrial function may result in deficient immunity.

## Circadian Rhythmicity, Metabolism, Intestinal Microbiota, and the Immune System. How Do These Interrelate?

The functional relationship between circadian rhythmicity and metabolism is widely recognized (Reinke and Asher, 2019), and so are the links between circadian rhythmicity and intestinal microbiota (Liang et al., 2015; Voigt et al., 2016) and between circadian rhythmicity and the immune system (Geiger et al., 2015; Lu et al., 2017; Scheiermann et al., 2018). In addition, there is strong evidence of crosstalk between metabolism and the immune response (Ganeshan and Chawla, 2014; Caputa et al., 2019) and between the intestinal microbiota and the immune response (Postler and Ghosh, 2017).

Fewer studies analyze the concurrent interaction between more than two of these biological domains, and when they do, they generally only include three, such as the crosstalk between the immune system, metabolism, and circadian rhythmicity (Early and Curtis, 2016; Carrol et al., 2019) or between intestinal microbiota, circadian rhythmicity, and metabolism (Parkar et al., 2019).

Based on the extensive literature on each of the individual biological domains referred to above as well as on their interactions, here we try to convey the view that the circadian clock, metabolism (both at the systemic and at the cellular level), intestinal microbiota, and immune system concurrently communicate with each other.

Moreover, based on recent findings on basic mitochondrial biology, as well as on the role of mitochondria in each of the four biological domains here mentioned, we propose that mitochondria regulate the back and forward traffic of information from one domain to the other.

Network theory has provided evidence that “a disease is rarely a consequence of an abnormality in a single gene, but reflects the perturbations of the complex intracellular network,” hence the concept of network medicine (Barabási et al., 2011). Perhaps, we should add that not only is a single gene not responsible for a given disease, but rather, not even only a single biological domain (circadian rhythmicity, etc.) is responsible, as different biological domains are tightly interdependent. Therefore, identifying a “hub” within the circadian clock-metabolism-intestinal microbiota-immune system network would help to understand its inner interactions better; we propose that mitochondria are such a “hub.”

**FIGURE 4:**
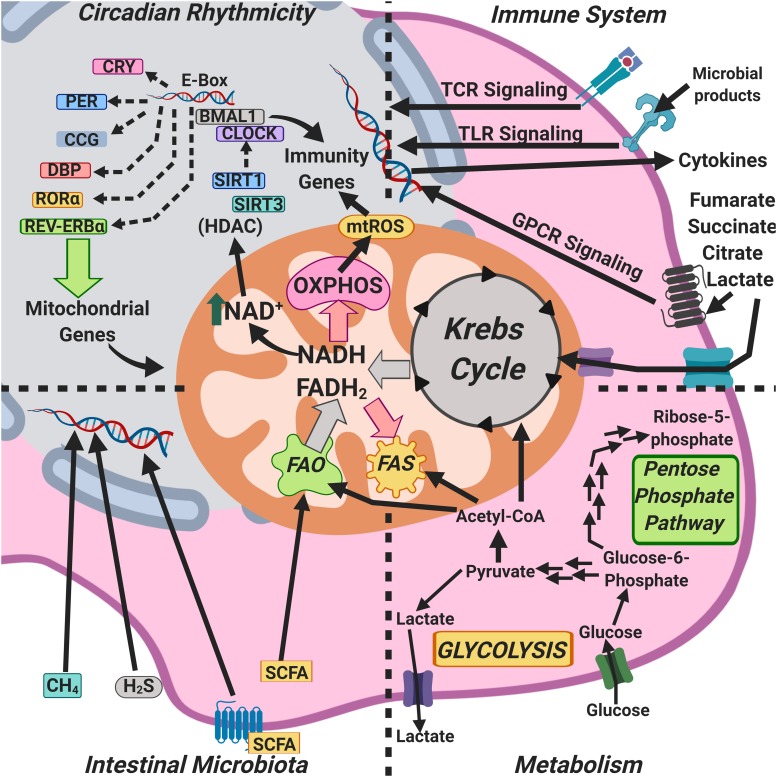
Mitochondria as an integrative hub coordinating circadian rhythms, metabolism, the microbiome, and immunity.

Mitochondria are central to energy homeostasis in eukaryotes and display a number of other functions that put them at the crossroads of multiple physiological functions. Of note, mitochondria produce signaling molecules and also receive and process signals from outside the mitochondria; hence, their proposed role as an “integrative center” (Figure 4).

Mitochondrial signals, including mROS, oxidized mtDNA, extracellular ATP, and mitochondrial membrane potential (ΔΨm) depolarization, all of which are hallmarks of dysfunctional mitochondria, activate the NLRP3 (nucleotide-binding domain and leucine-rich repeat pyrin 3 domain) inflammasome (Próchnicki and Latz, 2017; Jackson and Theiss, 2019). In addition, in inflammatory pathologies such as inflammatory bowel disease (IBD), mitochondrial DNA can be released into the serum and be recognized as a DAMP, contributing to a more systemic inflammatory process (Boyapati et al., 2018). IL-10 deficiency in IBD patients and in mouse models of colitis leads to the accumulation of damaged mitochondria and, as a result, to the activation of the NLRP3 inflammasome in macrophages (Ip et al., 2017).

The Parkinson’s disease-associated mitochondrial serine protease HtrA2 downregulates the activity of NLRP3 and absent in melanoma 2 (AIM2) inflammasomes by preventing the sustained accumulation of the inflammasome adaptor apoptosis-related speck-like protein containing a CARD (ASC), and thus, HtrA2 acts as a mitochondrial quality control element that keeps NLRP3 and AIM2 inflammasomes tightly controlled (Rodrigue-Gervais et al., 2018), thus linking mitochondria and inflammation. Loss of HtrA2 increases the number of damaged mitochondria as well as the levels of unfolded respiratory chain subunits, indicating that HtrA2 is an important component in the maintenance of proteostasis in the mitochondrial inter-membrane space. On the other hand, the levels of HtrA2 increase in response to several stressors (Moisoi et al., 2009; Baker et al., 2011).

The circadian clock generates NAD^+^-dependent oscillations in the oxidative capacity of mitochondria through the rhythmic transcription of nicotinamide phosphoribosyl-transferase, ensuring oxidative rhythms compatible with the fasting-feeding cycle, and maximizing energy production and consumption, and, thus, NAD^+^ couples circadian rhythms and metabolism (Peek et al., 2013; Figure 4). On the other hand, the concentration of NAD^+^ in mitochondria regulates the activity of the sirtuin deacetylase-3 (SIRT3), which in turn controls the acetylation and activity of other key metabolic enzymes. In most cases, acetylation reduces the enzymatic activity of modified mitochondrial proteins, presumably impairing mitochondrial metabolism and, by removing acetyl moieties from protein substrates, SIRT3 would restore their activity (Peterson et al., 2018).

The circadian regulator CLOCK has an intrinsic acetyl-transferase activity, which enables circadian-dependent chromatin remodeling by acetylating histones and non-histone proteins; the CLOCK acetyl-transferase activity is counterbalanced by the deacetylase SIRT1, whose activity is dependent on intracellular NAD^+^ levels (Nakahata et al., 2009; Figures 1, 4). Likewise, the circadian oscillation of NAD^+^ levels drives the activities of at least three sirtuins, SIRT1, SIRT3, and SIRT6, regulating glucose, cholesterol, and fatty acid metabolism, among other metabolic functions (Imai and Guanrente, 2016).

The concentrations of a wide array of mitochondria-associated metabolites, such as NAD^+^, ATP, mROS, and Krebs cycle intermediates are dependent on circadian rhythmicity; notable among them are components of the pyruvate dehydrogenase complex, which catalyzes the rate-limiting step in mitochondrial carbohydrate metabolism, and the carnitine palmitoyl-transferase 1, the rate-limiting enzyme in the transport of fatty acids into the mitochondrial matrix (Neufeld-Cohen et al., 2016). In addition to clock-controlled concentration of mitochondrial enzymes, mitochondrial respiration is also strongly influenced by the molecular circadian clock (Scrima et al., 2016) and by nutrition type (Neufeld-Cohen et al., 2016), thus linking mitochondrial function, metabolism, and circadian rhythmicity.

Nutrition type is a key determinant of intestinal microbiota composition, and the intestinal microbiota play an important role in the anatomical development of the intestine to sustain such microbiota (Moreira-Rosário et al., 2019). Crypt formation in the intestine is dependent on the abundance and function of mitochondria, since mitochondrial dysfunction impairs the ability of the intestinal stem cells (ISCs), which have a large number of mitochondria, to produce ATP, altering their self-renewal and differentiation (Berger et al., 2016). Upon colonization, intestinal microbiota produce SCFAs, and these activate the peroxisome proliferator-activated receptor γ (PPARγ) co-activator isoform α (PGC1α), a master regulator of mitochondrial biogenesis and function and thus, cell stimulation with SCFAs results in glucose uptake, oxidative phosphorylation, fatty acid β-oxidation, and mitochondrial biogenesis (Belizário et al., 2018; Figure 4).

Intestinal microbiota-derived butyrate induces mitochondrial fusion (Mollica et al., 2017) and a phase shift in the expression of the clock genes BMAL1 and Per2 (Leone et al., 2015). Methane, another intestinal microbiota product, can freely diffuse across cell membranes and thus reach mitochondria; the exogenous administration of methane in ischemia-reperfusion increases oxidative phosphorylation, which could indicate a bioactive role in mitochondria, thus linking intestinal microbiota with mitochondrial function and circadian rhythmicity (Strifler et al., 2016).

Loss of mitochondrial quality control impairs autophagy, exacerbates inflammatory processes, and increases cell death rates, with multiple physiological consequences (Baixauli et al., 2015; Demers-Lamarche et al., 2016).

Altogether, any tentative answer to the question raised in the previous heading, “How do these interrelate?” points to mitochondria.

## Concluding Remarks

We have tried to convey the view that circadian clocks, metabolism, intestinal microbiota, and the immune response tightly interact with each other and that their regulatory mechanisms seem to converge in mitochondria.

Outlining the mechanisms that regulate circadian clocks, metabolism, intestinal microbiota, and immune response communication may help to understand health-disease processes better, and, on the other hand, defining how mitochondria receive, process, and respond to signals from each one of these four biological domains, as well as the chemical nature of those signals, will improve our understanding of mitochondria.

## Author Contributions

BA-L wrote the manuscript and designed and prepared the figures. MM-A, HD, and MD reviewed and wrote specific parts of the manuscript. JS-G conceived the review, wrote the manuscript, and designed the figures.

## Conflict of Interest

The authors declare that the research was conducted in the absence of any commercial or financial relationships that could be construed as a potential conflict of interest.
